# The effects of professional expertise on perceptions of treatment need in patients with class II division 1 malocclusion: a comparison between orthodontists, general dentists, and lay people in Germany

**DOI:** 10.1007/s00056-024-00551-0

**Published:** 2024-11-04

**Authors:** Sarah Bühling, Stefanie Neidhardt, Babak Sayahpour, Sara Eslami, Nicolas Plein, Stefan Kopp

**Affiliations:** Department of Orthodontics, Johann-Wolfgang Goethe University, Theodor-Stern-Kai 7, 60596 Frankfurt, Germany

**Keywords:** Malocclusion, Angle class II division 1, Esthetics, Facial asymmetries, Index of orthodontic treatment need, Malokklusion, Angle-Klasse-II-Division‑1, Ästhetik, Gesichtsasymmetrien, Kieferorthopädische Indikationsgruppen

## Abstract

**Purpose:**

This study aimed to compare the perception of practitioners with varying levels of expertise and laypeople regarding the orthodontic treatment need and facial harmony in patients with increased anterior overjet.

**Methods:**

Three groups of observers (orthodontists, general dentists, and laypeople, in total *n* = 48) were asked to rate on images—using a 10-point visual analog scale (VAS)—the facial harmony and treatment need of a sample of 8 patients with class II division 1 malocclusion and overjets of 2, 4, 6, and 8 mm.

**Results:**

Statistically significant differences were observed between the three groups of observers regarding patients with an overjet of 4 mm and above (*p* < 0.001). Treatment need was perceived at an overjet of 4 mm by orthodontists and 6 mm by general dentists, whereas laypeople did not perceive a need for treatment in any of the groups (*p* < 0.001). Regarding perception of facial harmony, orthodontists had the lowest threshold (4 mm overjet), while dentists followed at a greater overjet of 6 mm or more (*p* < 0.001). A statistically significant correlation between the noticed facial harmony and the perceived orthodontic treatment need was found in all observers (*p* < 0.001).

**Conclusion:**

The perceived orthodontic treatment need for class II division 1 malocclusion increased with increasing professional expertise.

## Abstract (de)

**Ziel:**

Diese Studie zielte darauf ab, die Wahrnehmung von Fachleuten mit unterschiedlichen Erfahrungsstufen und Laien in Bezug auf den kieferorthopädischen Behandlungsbedarf und die Gesichtsästhetik bei Patienten mit vergrößerter sagittaler Frontzahnstufe zu vergleichen.

**Methoden:**

Drei Gruppen von Beobachtern (Kieferorthopäden, Allgemeinzahnärzte und Laien, insgesamt *n* = 48) wurden gebeten, auf Bildern die Gesichtsästhetik und den Behandlungsbedarf einer Stichprobe von 8 Patienten mit einer Klasse-II-Division-1-Malokklusion und einem Overjet von 2, 4, 6 und 8 mm anhand einer visuellen Analogskala (VAS) mit 10 Punkten zu bewerten.

**Ergebnisse:**

Es wurden statistisch signifikante Unterschiede zwischen den 3 Beobachtergruppen bei Patienten mit einem Overjet von 4 mm und mehr festgestellt (*p* < 0,001). Der Behandlungsbedarf wurde von Kieferorthopäden bei einem Overjet von 4 mm und von Allgemeinzahnärzten bei einem Overjet von 6 mm wahrgenommen, während Laien in keiner der Gruppen einen Behandlungsbedarf erkannten (*p* < 0,001). In Bezug auf die Wahrnehmung der Gesichtsästhetik hatten Kieferorthopäden die niedrigste Schwelle (4 mm Overjet), während Zahnärzte bei einem größeren Overjet von 6 mm oder mehr folgten (*p* < 0,001). Eine statistisch signifikante Korrelation zwischen der wahrgenommenen Gesichtsästhetik und dem wahrgenommenen kieferorthopädischen Behandlungsbedarf wurde bei allen Beobachtern festgestellt (*p* < 0,001).

**Schlussfolgerung:**

Der wahrgenommene kieferorthopädische Behandlungsbedarf bei einer Klasse-II-Division-1-Malokklusion stieg mit zunehmender fachlicher Expertise.

## Introduction

Hallmark of class II division 1 malocclusions is the increased anterior overjet, which is accompanied with lip incompetency in more severe cases [1]. The patients are at risk of several health issues, including traumatic damage to the maxillary incisors, mouth breathing, which increases the risk of gingivitis and caries, and a higher likelihood of developing craniomandibular disorders [2–8]. Thus, reducing the increased anterior overjet in these malocclusions is a primary objective of orthodontic treatment.

Orthodontic indices with different cut-off values have been developed to assess the orthodontic treatment need of different malocclusions. While some indices focus solely on dental, occlusal, and functional factors, others also consider esthetic perception [9].

Since 2002, the German public health insurance system has utilized the Orthodontic Indication Group (KIG) system to determine the need for orthodontic treatment [9]. This system is focused exclusively on the dental malocclusion aspect and does not account for esthetic or functional considerations. Under this system, public insurance coverage for treatment costs in the class II malocclusion category is only provided for overjet values greater than 6 mm.

Despite the KIG system’s sole emphasis on dental malocclusion aspects, previous studies have demonstrated that orthodontists and general dentists consider both functional and esthetic factors when determining orthodontic treatment need [10–12]. On the other hand, laypeople tend to prioritize the esthetic aspect of malocclusions [11, 13–17].

Generally, facial symmetry as well as white and straight teeth are considered esthetically pleasing from the laypeople perspective [13, 18]. However, improvement of oral function does not necessarily contribute to the patient’s satisfaction [14, 17]. Orthodontic treatment need is not deemed necessary by laypeople as long as there is no crowding or spacing of teeth or only a large overjet and as long as there are no symptoms of temporomandibular disorder (TMD) [16, 17]. Thus, understanding of patients’ perception of facial harmony and orthodontic treatment need is crucial to ensure their satisfaction.

Understanding the perception of general dentists is also crucial, as they are important referral sources for orthodontists in the German healthcare system. They often identify the need for orthodontic treatment and refer patients to orthodontic offices during routine check-ups [16, 17].

Achieving a consensus among patients, general dentists, orthodontists, and healthcare providers regarding the need for orthodontic treatment would ensure a high level of healthcare quality and patient satisfaction [13, 14]. Conversely, disagreements on this topic may compromise the quality of care and result in patient dissatisfaction.

Therefore, the present study aimed to determine the differences in the perception of orthodontic treatment need and facial harmony of patients with different severities of increased anterior overjet from the perspective of orthodontists, general dentists and laypeople. Secondary aim was to determine whether there is a correlation between perceived facial harmony and the perceived treatment need.

## Materials and methods

### Study design and ethic approval

Ethic approval for this monocentric cross-sectional survey study was granted by the Institutional Review Board of Goethe University of Frankfurt am Main (no. 2024-1990). Informed consents were obtained from the patients or their legal guardians regarding publishing their data and photographs.

### Study groups, sample size calculation

Three groups were included in this study: orthodontists, general dentists, and laypeople. Sample size was calculated with the probability of error set at α = 5% and a power of 80%. For the determination of the effect size, it was assumed that the Mann–Whitney estimator for the comparison of the score values of laypeople to general dentists was approximately 0.7.

In addition, for the Mann–Whitney estimator, a value of 0.8 was assumed for the comparison of laypeople to orthodontists and a value of 0.65 for the comparison of the general dentists and the orthodontists.

Therefore, a sample size of 48 (16 participants per group) was calculated.

### Participant recruitment

In all, 48 adult participants (16 orthodontists, 16 general dentists, and 16 laypeople with no experience in dentistry) with Caucasian ethnicity living in Germany were recruited. Their demographic information is shown in Table 1.

**Table 1:** Participants’ descriptive data Deskriptive Daten der Teilnehmer

		Orthodontists		General dentists		Laypeople	
		*N*	%	*N*	%	*N*	%
Age, years	31 ≤ 41	1	6.25	9	56.25	8	50.0
	41 ≤ 51	9	56.25	5	31.25	4	25.0
	51 ≤ 61	4	25.0	1	6.25	1	6.25
	61 <	2	12.5	1	6.25	3	18.75
Gender	Male	9	56.25	10	62.5	8	50.0
	Female	7	43.75	6	37.5	8	50.0

### Sample images and questionnaire preparation

**Fig. 1:**
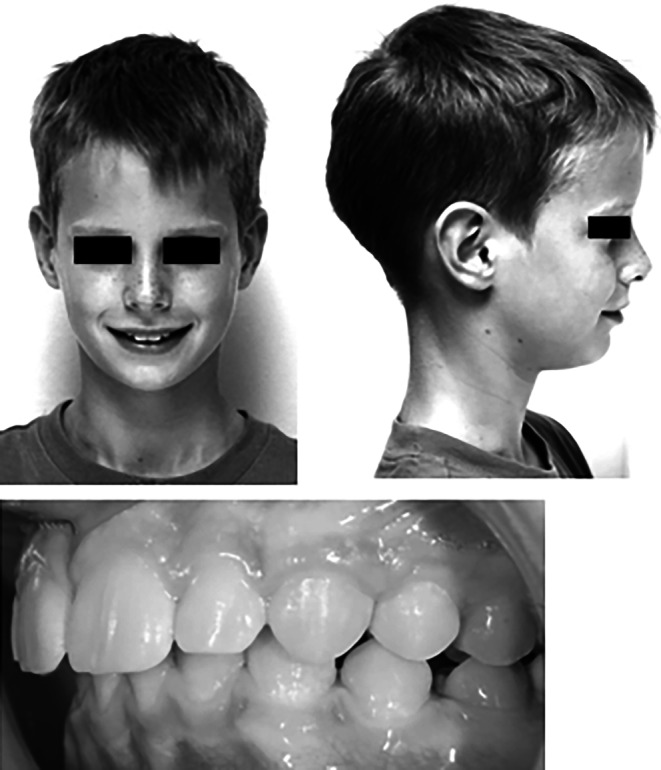
Sample image of a patient with increased overjet Beispielbild eines Patienten mit vergrößerter sagittaler Frontzahnstufe

**Fig. 2:**
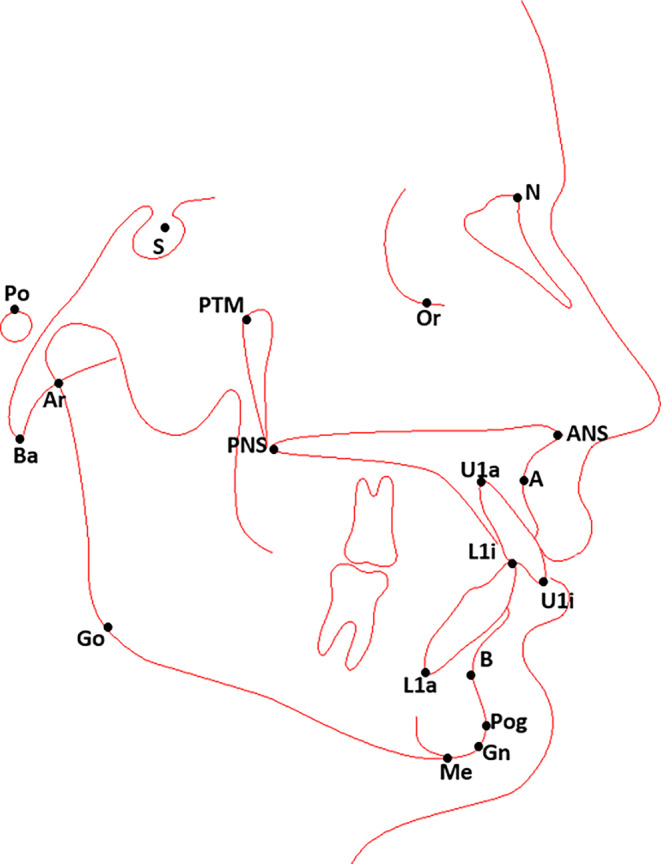
Tracing of the lateral cephalogram. Following landmarks were used for the analysis: *S* sella turcica, *N* nasion, *Ba* basion, *Or* orbitale, *P* porion, *ANS* anterior nasal spine, *PTM* pterygomaxillary fissure, *A* A point, *B* B point, *U1a* upper incisor apex, *U1i* upper incisor edge, *L1a* lower incisor apex, *L1i* lower incisor edge, *Me* menton, *Gn* gnathion, *Ar* articulare, *Go* gonion Durchzeichnung des Fernröntgenseitenbildes. Folgende Referenzpunkte wurden für die Analyse verwendet: *S* Sella turcica, *N* Nasion, *Ba* Basion, *Or* Orbitale, *P* Porion, *Spa* Spina nasalis anterior, *Pm* Pterygomaxillare, *A* A-Punkt, *B* B-Punkt, *Isa* Incision superius apicale, *Is* Incision superius, *Iia* Incision inferius apicale, *Ii* Incision inferius, *Me* Menton, *Gn* Gnathion, *Ar* Articulare, *Go* Gonion

Sample images (Fig. 1) consisted of the two extra-oral photographs (smiling frontal view and profile at rest) as well as one intra-oral image of the occlusion (left side). They were taken from 4 boys and 4 girls who fulfilled the following inclusion criteria:

- Patients undergoing orthodontic therapy in the orthodontic department,
- Below 18 years of age,
- Anterior overjet of 2, 4, 6, and 8 mm,
- Skeletal class II division 1 malocclusion (ANB > 6°, U1-NA > 22°; Fig. 2),
- Lack of any visible crowding, irregularity or spacing,
- Lack of midline deviations of 0.5 mm or higher,
- Fully permanent dentition,
- Caucasian ethnicity, and
- Available and complete pretreatment diagnostic records including lateral cephalograms.

Patients with syndromes manifesting on the face, visible facial scars, missing anterior teeth, discolorations, braces, and large fillings or crowns on the anterior teeth and other malocclusions such as crossbite or anterior openbite were excluded.

**Fig. 3:**
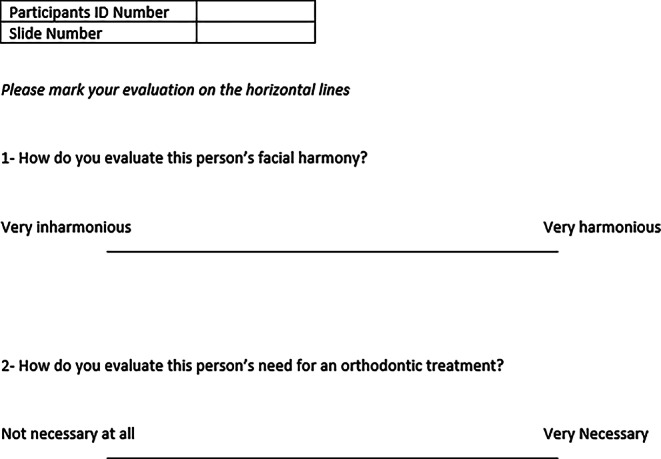
Questionnaire Fragebogen

The images were converted to black and white, printed on photographic paper and assembled into a slide containing a visual analog scale (VAS)-based questionnaire (Fig. 3), containing two questions:

1. How do you rate this person’s facial harmony? (Left: very inharmonious, right: completely harmonious)
2. How do you rate this person’s need for an orthodontic treatment? (Left: not necessary at all, right: very necessary)

### Observers’ rating of sample images

Participants were informed that they would be presented with several images and instructed to rate them on the VAS scale based on their perception of facial harmony and treatment necessity of each model using a pen with a fine 0.7 mm tip width (Fig. 3). The pictures were presented in a random manner to the participants and they received 1 min for evaluation of each patient.

Before conducting the study, four experienced orthodontists independently scored the images. One month later, the same orthodontists re-evaluated the images, and they reached a unanimous agreement that aligned with their initial assessments. This demonstrated a high level of consistency in image scoring, rendering reliability testing unnecessary.

### VAS score analysis

All questionnaires were analyzed by one examiner. Answers on the 100 mm VAS score were analyzed using a standard metric ruler with a precision of 0.5 mm. The score was rounded to the next whole millimeter. A score ≥ 5 meant that the participant considered that orthodontic treatment for this patient was necessary or that the face was seen as harmonious [19].

### Statistical analysis

BiAs software version 11.06 (BiAS for Windows; Epsilon Verlag GmbH, Frankfurt, Germany) was used for the statistical analysis of this study. The statistical analysis was done by the Institute for Biostatistics and Mathematical Modeling at Johann Wolfgang Goethe University in Frankfurt am Main. The nonparametric Kruskal–Wallis test was used to examine the differences between the groups. The Χ^2^ consistency table test was used to show possible relationships of the categorical data. If the test was weak, the Craddock–Flood Χ^2^ test was used. Spearman’s rank correlation was used to quantify the association between two subjects (orthodontic treatment need, facial harmony). The confidence level was set at α = 5% and the power at 80%.

## Results

### Perception of orthodontic treatment need

**Fig. 4:**
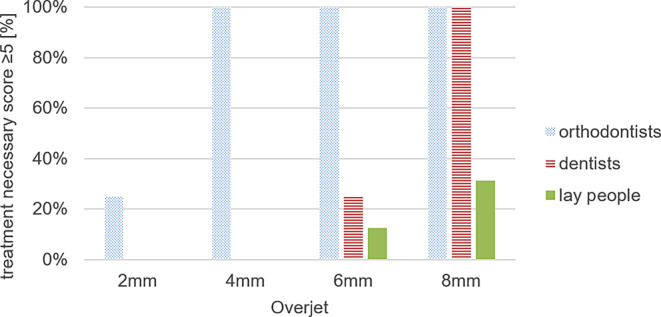
Perceived treatment need depending on stage of overjet. Percentage of all ratings with a score ≥ 5 are shown for each group (orthodontists, dentists, laypeople) Wahrgenommener Behandlungsbedarf in Abhängigkeit vom Stadium des Overjet. Der Prozentsatz aller Bewertungen mit einem Wert ≥ 5 wird für jede Gruppe (Kieferorthopäden, Zahnärzte, Laien) dargestellt

Tables 2 and 3 as well as Fig. 4 display the mean values of perceived treatment need VAS score ratings by participants for each patient.

**Table 2:** Perception of orthodontic treatment need in each group of patients by participants. Visual analog scale (VAS) scores are shown by their mean value, standard deviation (SD), minimum (Min), maximum (Max) Wahrnehmung des kieferorthopädischen Behandlungsbedarfs bei den einzelnen Patientengruppen durch die Teilnehmer. Gezeigt werden VAS(visuelle Analogskala)-Bewertungen mit Mittelwert, Standardabweichung (SD), dem niedrigsten (Min) und dem höchsten Wert (Max)

Patient’s overjet	Group	Mean	SD	Min	Max	*p* value^a^
2 mm	Orthodontists	3.85	2.49	0.5	9.3	0.122
	General dentists	2.21	1.71	0.0	4.3	
	Laypeople	2.42	1.37	0.6	4.1	
4 mm	Orthodontists	7.36	1.24	5.9	8.9	< 0.001
	General dentists	2.35	1.36	0.0	3.4	
	Laypeople	2.78	1.37	1.4	4.8	
6 mm	Orthodontists	7.87	1.31	6.2	10.0	< 0.001
	General dentists	6.54	2.26	4.0	9.6	
	Laypeople	3.22	2.05	1.3	5.6	
8 mm	Orthodontists	8.60	1.22	7.0	10.0	< 0.001
	General dentists	8.21	1.68	6.0	10.0	
	Laypeople	4.40	2.51	0.8	7.8	

^a^ *p* value of Kruskal–Wallis test. Statistical significance is at *p* ≤ 0.001

**Table 3:** Frequencies of the dichotomized visual analog scale (VAS) score regarding the perception of treatment need. Score summarizes the number of ratings greater and equal to or less than 5 Häufigkeiten der dichotomisierten VAS(visuelle Analogskala)-Bewertung bezüglich des wahrgenommenen Behandlungsbedarfs. Der Score fasst die Anzahl der Bewertungen zusammen, die höher oder gleich bzw. niedriger als 5 sind

Patient’s overjet	VAS score	Orthodontists		General dentists		Laypeople		*p* value^c^
		*N*	%	*N*	%	*N*	%	
2 mm	≥ 5	4	25.0	0	0	0	0	< 0.01^a^
	< 5	12	75.0	16	100	16	100	
4 mm	≥ 5	16	100	0	0	0	0	0.00^b^
	< 5	0	0	16	100	16	100	
6 mm	≥ 5	16	100	12	75.0	2	12.5	0.00^b^
	< 5	0	0	4	25.0	14	87.5	
8 mm	≥ 5	16	100	16	100	5	31.25	< 0.01^a^
	< 5	0	0	0	0	11	68.75	

^a^ *p*-value of Craddok–Flood test

^b^ Χ^2^-contingency table test

^c^ Statistical significance is *p* ≤ 0.001

No statistically significant differences were found in the ratings of patients with an overjet of 2 mm (*p* = 0.122). None of the participants recognized a need for orthodontic treatment in patients with an anterior overjet of 2 mm, although the orthodontist group showed notable discrepancies in their assessments of the 2 mm overjet.

However, statistically significant differences were recorded at overjets of 4, 6, and 8 mm between the groups (*p* < 0.001).

The first treatment need was identified by orthodontists at the overjet of 4 mm with a mean VAS score of 7.36, while neither dentists nor laypeople recognized the need for treatment at this overjet.

The treatment need of patients with an overjet of 6 mm was recognized by both general dentists (mean value 6.54) and orthodontists (mean value 7.87).

Interestingly, orthodontists’ perceived treatment need did not increase with the severity of malocclusion. In contrast to general dentists and orthodontists, 87.5% of laypeople did not perceive any treatment need at the overjet of 6 mm, and the highest VAS score in this regard was only 5.6.

The orthodontists and general dentists were in agreement that an overjet of 8 mm required treatment, as indicated by their mean VAS scores of 8.60 and 8.21, respectively. However, there was considerable variability among laypeople’s ratings of patients with an overjet of 8 mm, ranging from a minimum VAS score of 0.8 to a maximum of 7.8. Despite this variability, the mean VAS score for laypeople in this group was 4.40, indicating that they did not perceive a treatment need.

### Perception of facial harmony

**Fig. 5:**
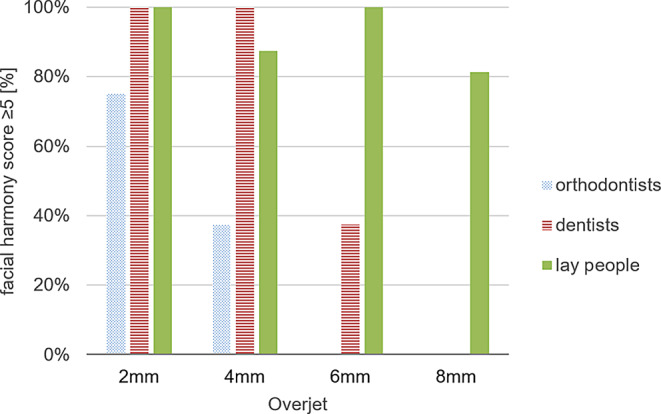
Perceived facial harmony depending on stage of overjet. Percentage of all ratings with a score ≥ 5 are shown for each group (orthodontists, dentists, laypeople) Wahrgenommene Gesichtsästhetik in Abhängigkeit vom Stadium des Overjet. Der Prozentsatz aller Bewertungen mit einem Wert ≥ 5 wird für jede Gruppe (Kieferorthopäden, Zahnärzte, Laien) dargestellt

Table 4 and Fig. 5 illustrate observers’ ratings regarding the patients’ facial harmony. Starting with an overjet of 4 mm, significant differences were recorded between the groups regarding the perceived facial harmony (*p* ≤ 0.001).

**Table 4:** Perception of facial harmony in each group of patients by participants. Visual analog scale (VAS) score are shown by their mean value, standard deviation (SD), lowest value (Min), highest value (Max) Wahrnehmung der Gesichtsästhetik bei den einzelnen Patientengruppen durch die Teilnehmer. Gezeigt werden die VAS(visuelle Analogskala)-Bewertungen mit ihrem Mittelwert, der Standardabweichung (SD), dem niedrigsten (Min) und dem höchsten Wert (Max)

Patient’s overjet	Group	Mean	SD	Min	Max	*p* value^a^
2 mm	Orthodontists	5.75	2.01	2.5	8.1	0.006
	General dentists	7.42	1.92	5.8	10.0	
	Laypeople	7.54	2.16	5.1	10.0	
4 mm	Orthodontists	4.48	2.31	1.6	5.9	< 0.001
	General dentists	7.71	1.49	5.2	9.8	
	Laypeople	7.27	1.77	4.7	9.3	
6 mm	Orthodontists	3.01	1.26	1.6	4.9	< 0.001
	General dentists	4.35	1.89	2.6	6.8	
	Laypeople	7.00	1.81	3.6	9.4	
8 mm	Orthodontists	1.92	1.19	0.3	3.5	< 0.001
	General dentists	2.82	1.49	0.2	4.3	
	Laypeople	6.53	2.25	4.3	9.6	

^a^ *p* value of Kruskal–Wallis test. Statistical significance is at *p* ≤ 0.001

Faces showing an overjet of 2 mm were mostly considered harmonious by the group of orthodontists (mean value 5.75) but some of them considered the face as inharmonious (minimum 2.5, maximum 8.1). Patients with an overjet of 4 mm or higher were regarded as inharmonious by the majority of orthodontists (4 mm overjet, mean value 4.48; 6 mm overjet, mean value 3.01; 8 mm overjet, mean value 1.92).

General dentists regarded faces with an overjet of 2 mm (mean value 7.42) and 4 mm (mean value 7.71) as harmonious. From an increase in the overjet to 6 mm, the faces were considered to be inharmonious (6 mm overjet, mean value 4.35; overjet of 8 mm, mean value 2.82).

Laypeople perceived all of the faces as harmonious regardless of the overjet (2 mm, mean value 7.54; 4 mm, mean value 7.27; 6 mm, mean value 7.0; 8 mm, mean value 6.53). However, a minimal drop in evaluations towards disharmony could be found with the increase of overjet (Table 5).

**Table 5:** Frequencies of the dichotomized visual analog scale (VAS) rating regarding the facial harmony. Score summarizes the number of ratings higher or lower than 5 Häufigkeiten der dichotomisierten VAS(visuelle Analogskala)-Bewertung bezüglich der Gesichtsästhetik. Der Score fasst die Anzahl der Bewertungen zusammen, die höher oder niedriger als 5 sind

Patient’s overjet	VAS score	Orthodontists		General dentists		Laypeople		*p* value^c^
		*N*	%	*N*	%	*N*	%	
2 mm	≥ 5	12	75.0	16	100	16	100	< 0.05^a^
	< 5	4	25.0	0	0	0	0	
4 mm	≥ 5	6	37.5	16	100	14	87.5	< 0.001^a^
	< 5	10	62.5	0	0	2	12.5	
6 mm	≥ 5	0	0	6	37.5	16	100	0.00^b^
	< 5	16	100	10	62.5	0	0	
8 mm	≥ 5	0	0	0	0	13	81.25	< 0.001^a^
	< 5	16	100	16	100	3	18.75	

^a^ *p*-value of Craddok–Flood test

^b^ Χ^2^-contingency table test

^c^ Statistical significance is *p* ≤ 0.001

### Correlation of facial harmony and treatment need

**Fig. 6:**
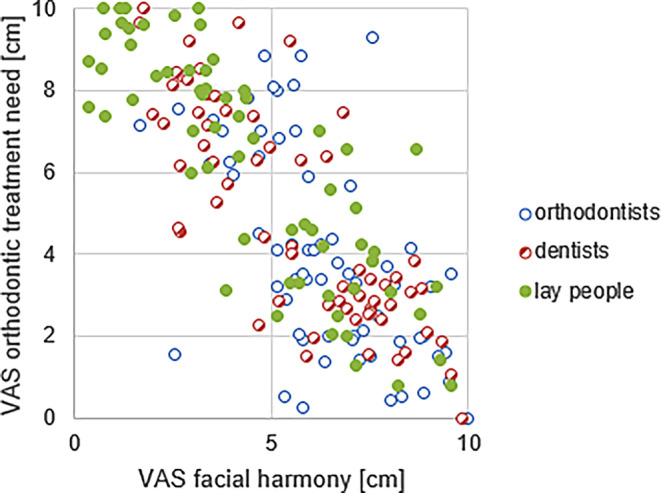
Correlation between facial harmony and perceived orthodontic treatment need. All ratings for each group (orthodontists, dentists, laypeople) are shown. *VAS* visual analog scale Korrelation zwischen Gesichtsästhetik und wahrgenommenem kieferorthopädischen Behandlungsbedarf. Alle Bewertungen für jede Gruppe (Kieferorthopäden, Zahnärzte, Laien) werden dargestellt. *VAS* visuelle Analogskala

To quantify the correlation between perceived orthodontic treatment need and considered harmony of the face, the Spearman’s rank correlation was used and a value of 0.662 was obtained with a *p*-value < 0.001. Consequently, the correlation was found to be statistically significant. With an increasing VAS for perceived treatment need, the VAS for harmony of the face decreased (Fig. 6). This correlation can be found in the ratings within all three groups.

## Discussion

The results of the present study showed significant differences between orthodontists, general dentists, and laypeople regarding their perception of treatment need and facial harmony in patients with class II division 1 malocclusions.

Differences in perception of treatment need in groups with different professional experience have been shown in previous studies and our findings confirm these results [20–24]. However, we have added the perception of facial harmony to our methodology, as the ideal image of facial and dental features may differ significantly between dental professionals and laypeople [20, 25]. Laypeople may recognize an increased overjet but still perceive the face as esthetically pleasing. There is still no accurate definition of a “beautiful face” as all people seem to have different points of view [10, 20, 26]. Nevertheless, a strong correlation was observed between the perception of facial harmony and treatment need by all participants. The increase in VAS rating for the perceived treatment need correlated with the decrease in VAS rating for facial harmony (Spearman’s rank correlation yielded a *p*-value < 0.001). These results are consistent with former studies that attempted to define standards for beautiful facial features [10, 20, 26].

Similar to other studies, orthodontists in our study displayed a more critical evaluation of the patient’s facial harmony and treatment need compared to general dentists and laypeople, as they identified a treatment need at an overjet of 4 mm [22, 27, 28]. It is noteworthy that this threshold is lower than the German public health insurance (KIG system) guideline, which considers a treatment indication at an overjet above 6 mm [29].

Previous studies have shown that orthodontists tend to have a more critical perception of orthodontic treatment needs, which can be attributed to their higher expertise and knowledge in the field of orthodontics [3, 6, 7]. Orthodontic specialists possess a more comprehensive understanding of the detrimental effects of an increased overjet and its accompanying lip incompetency on dental and oral health, such as risk of trauma and development of temporomandibular disorders [2, 4, 5]. Kuroda et al. [12] suggest that the recognition of need for orthodontic treatment of an enlarged overjet increases with increasing experience in the field of orthodontics.

Our results showed that laypeople did not perceive a need for orthodontic treatment or lack of facial harmony in patients with increased overjet, even though they registered the worsening of the overjet. It is important to note that the patients showed straight teeth and lacked visible irregularities or spacings. Angle class II malocclusion has previously been determined as esthetically pleasing in laypeople’s eyes [30]. This was also confirmed in the present study and underlines the laypeople’s focus on esthetic aspects of the malocclusion as opposed to the functional aspects [16, 31]. Previous studies have defined when there is a general need for orthodontic treatment by comparing various malocclusions of the teeth [24, 32]. However, the bases for the decisions of the individual respondents were not specified in these studies. Thus, it was never made clear as to the basis of which malocclusions of the teeth the decision for a necessary treatment was made [33, 34]. In the study by Prahl-Andresen et al. [35], profile drawings and color photographs of dentures were evaluated by orthodontists, general dental practitioners, and parents. The parents found significantly more of the photographs to be acceptable, whereas the opinions of the orthodontists and general dental practitioners differed only for certain malocclusions of the teeth; both groups rated more photographs as unacceptable than the parents’ group. Dentists often assess the need for treatment here as necessary only when the malocclusion feature is more pronounced and so are slightly less sensitive compared to orthodontists [35, 36]. Kokich et al. [36] compared the opinions of orthodontists, general dental practitioners, and laypeople on the minimal changes acceptable in the shape, size, and position of the anterior teeth and surrounding soft tissue; they concluded that the orthodontists were very sensitive to changes from the ideal, while the laypeople did not always detect them, even in the presence of major changes. These observations also confirm the results of this study.

Furthermore, patients do not seem to be aware of the complex interrelationships of an enlarged overjet and symptoms linked to craniomandibular disorders (CMD) [21, 37].

Therefore, it is vital to create awareness about the topic from the patient’s point of view. General dentists have an essential role in this regard, being the primary contact and referral source for orthodontic treatment in the German healthcare system.

Compared to the orthodontists, general dentists showed a higher threshold for treatment need and identification of lack of facial harmony. They considered patients with an overjet of 6 mm or larger to be in need of treatment, which is in line with the guidelines of the KIG system. This could be explained through the dental curriculum in Germany. Classification of orthodontic treatment need based on KIG system is integrated in the dental curriculum of undergraduate orthodontic program in Germany and, thus, shapes the dental students’ perception of malocclusions’ severity. This could potentially lead to a diagnostic bias in general dentists, which would compromise their ability in detection and referral of patients with malocclusions below KIG threshold values. However, further studies are required to provide clarity on this assumption of bias in orthodontic diagnosis of general dentists in Germany.

Laypeople are not familiar with the functional aspects that orthodontists consider in deciding treatment needs. The lack of awareness in laypeople combined with general dentists’ failure to identify orthodontic treatment needs in certain patients can pose a risk of undiagnosed conditions like upper incisors’ susceptibility to traumatic injury and higher risk of developing CMD symptoms [12, 38]. Even though the evidence regarding the etiological role of dental malocclusions in development of CMD remains controversial and a recent literature review reported the absence of an association for the majority of occlusal traits [39], the source of the controversy appears to lay in the lack of standardization and partly insufficient scientific rigor of heterogeneous methods of CMD diagnosis [40, 41]. Since CMD can severely affect the quality of life, creating awareness about occlusion’s importance at a young age is crucial [7, 37, 42].

## Limitations

The authors acknowledge that different factors such as ethnicity, age, and personal preferences can influence one’s perception of facial attractiveness. To minimize these effects, we used faces of the same ethnic background and presented them in black and white. However, individual differences remain. Although the use of digitally manipulated photos of one single patient instead of images of different patients would have made the model photos more homogeneous, real patient pictures were used to avoid the artificial appearance associated with digitally manipulated images. This precaution was taken to mitigate any potential negative impact on observers’ perceptions caused by the presence of manipulated photos.

The use of a visual analog scale allowed for more accurate analysis of data but had the potential for variation in the interpretation of the scale. The study also recognizes the potential bias introduced by the Hawthorne effect and the exclusion of certain subjects.

## Conclusion

This study showed that orthodontic treatment need and facial harmony are evaluated differently by orthodontists, general dentists, and laypeople. While orthodontists perceived a treatment need for patients with an overjet of 4 mm or larger, the general dentists perceived a treatment need at an overjet of 6 mm or more, which corresponds with German KIG system.

In absence of incisor crowding or irregularities, laypeople’s perception of treatment need or facial inharmony was not significantly affected by the increase in overjet.

The results of the present study underline the importance of patient education regarding the functional aspects of malocclusions with increased anterior overjet and suggest the possible presence of bias in diagnostic abilities of general dentists in Germany.

## Data Availability

The data that support the findings of this study are available on request from the corresponding author (SB).

## References

[1] Bauss O, Freitag S, Röhling J, Rahman A (2008) Influence of overjet and lip coverage on the prevalence and severity of incisor trauma. J Orofac Orthop 69:402–410 19169637 10.1007/s00056-008-8805-1

[2] Rueda-Ibarra V, Scougall-Vilchis RJ, Lara-Carrillo E, Lucas-Rincón SE, Patiño-Marín N, Martínez-Castañon GA, Romero-Martínez M, Medina-Solis CE, Maupomé G (2022) Traumatic dental injuries in 6 to 12 years old schoolchildren: a multicenter cross-sectional study in Mexico. Braz oral res 36:e123. 10.1590/1807-3107bor-2022.vol36.0123 36228222 10.1590/1807-3107bor-2022.vol36.0123

[3] Stahl F, Grabowski R (2004) Malocclusion and caries prevalence: is there a connection in the primary and mixed dentitions? Clin Oral Invest 8:86–90 10.1007/s00784-003-0244-1 14691677

[4] Macey R, Thiruvenkatachari B, O’Brien K, Batista KBSL (2020) Do malocclusion and orthodontic treatment impact oral health? A systematic review and meta-analysis. Am J Orthod Dentofacial Orthop 157:738–44.e10 32487303 10.1016/j.ajodo.2020.01.015

[5] Yonezu T, Kojima T, Kumazawa K, Shintani S (2013) Longitudinal investigation of relationship between developmental changes in sagittal occlusion and caries in lower first permanent molars. Bull Tokyo Dent Coll 54:209–213 24521546 10.2209/tdcpublication.54.209

[6] Selaimen CMP, Jeronymo JCM, Brilhante DP, Lima EM, Grossi PK, Grossi ML (2007) Occlusal risk factors for temporomandibular disorders. Angle Orthod 77:471–477 17465655 10.2319/0003-3219(2007)077[0471:ORFFTD]2.0.CO;2

[7] Sonnesen L, Bakke M, Solow B (1998) Malocclusion traits and symptoms and signs of temporomandibular disorders in children with severe malocclusion. Eur J Orthod 20:543–559 9825557 10.1093/ejo/20.5.543

[8] Prodinger-Glöckl D (2021) Craniomandibuläre Dysfunktion erkennen und behandeln. Z Komplementärmedizin 13:43–47

[9] Brook PH, Shaw WC (1989) The development of an index of orthodontic treatment priority. Eur J Orthod 11:309–320 2792220 10.1093/oxfordjournals.ejo.a035999

[10] Birkeland K, Katle A, Løvgreen S, Bøe OE, Wisth PJ (1999) Factors influencing the decision about orthodontic treatment. A longitudinal study among 11- and 15-year-olds and their parents. J Orofac Orthop 60:292–307 10546413 10.1007/BF01301243

[11] Gosney MB (1986) An investigation into some of the factors influencing the desire for orthodontic treatment. Br J Orthod 13:87–94 3456796 10.1179/bjo.13.2.87

[12] Kuroda S, Fuji A, Sugie M, Uoi S, Kondo R, Ando R, Yamashiro T (2010) Relationship between orthodontic expertise and perception of treatment needs for maxillary protrusion: comparison of dental students, residents, and orthodontists. Am J Orthod Dentofacial Orthop 137:340–345 20197170 10.1016/j.ajodo.2008.04.029

[13] Sampson A, Jeremiah HG, Lai NN, Kirschen R (2022) The development of a guide to borderline orthodontic need. Prog Orthod 23(1):13. 10.1186/s40510-022-00407-6 35434773 10.1186/s40510-022-00407-6 PMC9013731

[14] Atik E, Turkoglu H (2023) Does different vertical position of maxillary central incisors in women with different facial vertical height affect smile esthetics perception? Prog Orthod 24(1):28. 10.1186/s40510-023-00479-y 37544965 10.1186/s40510-023-00479-y PMC10404574

[15] Motamedian SR, Najary S, Nikakhtar H, Rezvani M, Safavi SM (2023) Comparison of pleasant and unpleasant smile characteristics in the perception of the laypeople in an Iranian population. Am J Orthod Dentofacial Orthop. 10.1016/j.ajodo.2023.04.022 37565945 10.1016/j.ajodo.2023.04.022

[16] Singh V, Hamdan A, Rock P (2012) The perception of dental aesthetics and orthodontic treatment need by 10- to 11-year-old children. Eur J Orthod 34:646–651 21808073 10.1093/ejo/cjr080

[17] de Sousa ET, da Silva BF, Maia FBM, Forte FDS, Sampaio FC (2016) Perception of children and mothers regarding dental aesthetics and orthodontic treatment need: a cross-sectional study. Prog Orthod 17:37 27747529 10.1186/s40510-016-0149-6 PMC5107559

[18] Kerosuo H, Hausen H, Laine T, Shaw WC (1995) The influence of incisal malocclusion on the social attractiveness of young adults in Finland. Eur J Orthod 17:505–512 8682167 10.1093/ejo/17.6.505

[19] Flores-Mir C, Major PW, Salazar FR (2004) Self-perceived orthodontic treatment need evaluated through 3 scales in a university population. J Orthod 31:329–334 (discussion 02) 15608349 10.1179/146531204225020644

[20] Hönn M, Göz G (2007) The ideal of facial beauty: a review. J Orofac Orthop 68:6–16 17238049 10.1007/s00056-007-0604-6

[21] Wedrychowska-Szulc B, Syryńska M (2010) Patient and parent motivation for orthodontic treatment—a questionnaire study. Eur J Orthod 32:447–452 20008018 10.1093/ejo/cjp131

[22] Pogrel MA (1991) What are normal esthetic values? J Oral Maxillofac Surg 49:963–969 1886024 10.1016/0278-2391(91)90060-y

[23] Phillips C, Trentini CJ, Douvartzidis N (1992) The effect of treatment on facial attractiveness. J Oral Maxillofac Surg 50:590–594 1593319 10.1016/0278-2391(92)90439-7

[24] Berk NW, Bush HD, Cavalier J, Kapur R, Studen-Pavlovich D, Sciote J, Weyant RJ (2002) Perception of orthodontic treatment need: opinion comparisons of orthodontists, pediatric dentists, and general practitioners. J Orthod 29:287–291 (discussion 77) 12444269 10.1093/ortho/29.4.287

[25] Langlois JH, Kalakanis L, Rubenstein AJ, Larson A, Hallam M, Smoot M (2000) Maxims or myths of beauty? A meta-analytic and theoretical review. Psychol Bull 126:390–423 10825783 10.1037/0033-2909.126.3.390

[26] Livas C, Delli K (2013) Subjective and objective perception of orthodontic treatment need: a systematic review. Eur J Orthod 35:347–353 22250076 10.1093/ejo/cjr142

[27] Porter JP (2004) The average African American male face: an anthropometric analysis. Arch Facial Plast Surg 6:78–81 15023793 10.1001/archfaci.6.2.78

[28] Phillips C, Tulloch C, Dann C (1992) Rating of facial attractiveness. Comm Dent Oral Epid 20:214–220 10.1111/j.1600-0528.1992.tb01719.x 1526107

[29] Schopf P (2001) Die kieferorthopädischen Indikationsgruppen. Berufsverband der Deutschen Kieferorthopäden

[30] Hönn M, Dietz K, Godt A, Göz G (2005) Perceived relative attractiveness of facial profiles with varying degrees of skeletal anomalies. J Orofac Orthop 66:187–196 15959632 10.1007/s00056-005-0445-0

[31] Abu Arqoub SH, Al-Khateeb SN (2011) Perception of facial profile attractiveness of different antero-posterior and vertical proportions. Eur J Orthod 33:103–111 20558590 10.1093/ejo/cjq028

[32] Graber LW, Lucker GW (1980) Dental esthetic self-evaluation and satisfaction. Am J Orthod 77:163–173 6928344 10.1016/0002-9416(80)90004-4

[33] Marques LS, Pordeus IA, Ramos-Jorge ML, Filogônio CA, Filogônio CB, Pereira LJ, Paiva SM (2009) Factors associated with the desire for orthodontic treatment among Brazilian adolescents and their parents. BMC Oral Health 9:34 20021649 10.1186/1472-6831-9-34 PMC2801664

[34] Taibah SM, Al-Hummayani FM (2019) Agreement and association between normative and subjective orthodontic treatment need using the index of orthodontic treatment need. J Orthodont Sci 8:1 10.4103/jos.JOS_87_18 PMC6416992 31001493

[35] Prahl-Andersen B, Boersma H, van der Linden FP, Moore AW (1979) Perceptions of dentofacial morphology by laypersons, general dentists, and orthodontists. J Am Dent Assoc 98:209–212 284066 10.14219/jada.archive.1979.0456

[36] Kokich VO, Kiyak HA, Shapiro PA (1999) Comparing the perception of dentists and lay people to altered dental esthetics. J Esthet Dent 11:311–324 10825866 10.1111/j.1708-8240.1999.tb00414.x

[37] Kopp S, Brunzel BG, Sebald WG, Langbein U, Graf H (2002) Funktionsbefunde im kraniomandibulären System bei Jugendlichen im Alter von 15–19 Jahren. Man Med 40:359–366

[38] Baldwin DC (1980) Appearance and aesthetics in oral health. Comm Dent Oral Epid 8:244–256 10.1111/j.1600-0528.1980.tb01296.x 6936127

[39] Manfredini D, Lombardo L, Siciliani G (2017) Temporomandibular disorders and dental occlusion. A systematic review of association studies: end of an era? J Oral Rehabil 44:908–923 28600812 10.1111/joor.12531

[40] Matos M, Radke J (2020) Does the Manfredini et al 2017 systematic review contribute to the science of occlusion as an etiology of TMD? Adv Dent Tech

[41] Gremillion HA (2006) The relationship between occlusion and TMD: an evidence-based discussion. J Evid Based Dent Pract 6:43–47 17138396 10.1016/j.jebdp.2005.12.014

[42] Lomas J, Gurgenci T, Jackson C, Campbell D (2018) Temporomandibular dysfunction. Aust J Gen Pract 47:212–215 29621862 10.31128/AFP-10-17-4375

